# The Rotavirus Vaccine Landscape, an Update

**DOI:** 10.3390/pathogens10050520

**Published:** 2021-04-26

**Authors:** Roberto Cárcamo-Calvo, Carlos Muñoz, Javier Buesa, Jesús Rodríguez-Díaz, Roberto Gozalbo-Rovira

**Affiliations:** 1 Department of Microbiology, School of Medicine, University of Valencia, Av. Blasco Ibañez 17, 46010 Valencia, Spain; rocarcal@alumni.uv.es (R.C.-C.); carlos.munoz@uv.es (C.M.); javier.buesa@uv.es (J.B.); 2 Instituto de Investigación INCLIVA, Hospital Clínico Universitario de Valencia, 46010 Valencia, Spain

**Keywords:** rotavirus, gastroenteritis, vaccine, intussusception, diarrhea

## Abstract

Rotavirus is the leading cause of severe acute childhood gastroenteritis, responsible for more than 128,500 deaths per year, mainly in low-income countries. Although the mortality rate has dropped significantly since the introduction of the first vaccines around 2006, an estimated 83,158 deaths are still preventable. The two main vaccines currently deployed, Rotarix and RotaTeq, both live oral vaccines, have been shown to be less effective in developing countries. In addition, they have been associated with a slight risk of intussusception, and the need for cold chain maintenance limits the accessibility of these vaccines to certain areas, leaving 65% of children worldwide unvaccinated and therefore unprotected. Against this backdrop, here we review the main vaccines under development and the state of the art on potential alternatives.

## 1. Background

Rotavirus (RV) infections are the leading cause of severe acute childhood gastroenteritis, accounting for the majority of diarrhea episodes among children under 6 years of age and representing the predominant cause of diarrhea mortality not only in humans but also in a wide variety of animal species [1]. Cases show higher incidence and severity between 3 and 24 months of age [2]. Although childhood diarrhea or gastroenteritis of viral etiology has been a recognized concern throughout world history, the first human RV-associated infection dates from 1973 [3]. In 2016, RV accounted for about 128,500 deaths worldwide, mainly in low income countries (LICs) [4]; however, the number of deaths caused by this virus has decreased significantly since the introduction of RV vaccines in vaccination programs from around 2008, at which time deaths amounted to 453,000 per year [5]. Other studies estimate that before vaccines were introduced, RV caused about 111 million episodes of gastroenteritis each year; 25 million doctor visits; two million hospitalizations; and an average of between 352,000 and 440,000 deaths per year [6]. RV replicates in the enterocytes of the intestinal tract, leading to acute gastroenteritis (AGE) and shedding of virus in the stool [7]. It is estimated that approximately half of RV-associated deaths occur in just four countries: India, Nigeria, Pakistan and the Democratic Republic of Congo [8].

Since 2006, The World Health Organization (WHO) has recommended including RV vaccines in vaccination programs in Europe and the Americas, extending this recommendation globally in 2009 [9]. By 2018, a total of 92 countries had introduced RV vaccines into their national immunization programs, and in another six countries they were introduced on a phased or regional basis. In the meantime, in countries with fewer resources, vaccine introduction is supported by GAVI (The Vaccine Alliance). Of 73 potential aid-receiving countries, 46 introduced the vaccine in 2019 and eight more have recently been selected for aid and are planning to introduce it in the future [10].

Although RV incidence is greater in LICs, the virus is not limited to these countries; indeed, its effect is felt worldwide. Before the introduction of vaccines in the European Union, for example, RV was responsible for about 3.6 million episodes of AGE, 700,000 visits to the doctor; 87,000 hospitalizations; and 231 deaths among children under 5 each year, although hospitalizations have been reduced by 90% since introduction of the vaccines [11]. In addition, it is estimated that most children have been infected at least once, even if undiagnosed, by the age of 5, indicating an underestimation of the health burden caused by this virus. Similarly, although deaths caused by RV are mostly concentrated in children, RV infections can also affect adults throughout their lives, albeit with mild or even asymptomatic infections [12].

RV is a genus of the *Reoviridae* family and includes only viruses that infect vertebrates (birds and mammals). RV have a segmented genome composed of 11 double-stranded RNA molecules with the 3′ and 5′ endings preserved. The genome codes for six structural proteins (VP1-VP4, VP6 and VP7) and for six non-structural proteins (NSP1-NSP6). All segments are monocistronic, except for segment 11 which codes for two non-structural proteins (NSP5 and NSP6) [13]. The viral particles are apparently spherical, between 70 and 100 nm in diameter, with an icosahedral capsid and without a lipid envelope [14]. Infectious particles are formed by three concentric layers of protein, a structure known as a triple layered particle (TLP). The inner capsid contains the VP2 protein surrounding the genome [15] and several molecules of the VP1 proteins (the viral RNA-dependent RNA polymerase) [16] and VP3 (protein with guanylyltransferase and methylase activity) [17]; the intermediate capsid are composed of VP6 and are responsible for the main RV classification. The outer capsid also consists of VP7 protein and VP4 spicules emanating from the surface of the virion [18]. Both external capsid proteins, VP7 and VP4, possess neutralization epitopes and play an important role in virus entry and infection of target cells. In the intestine, the surface spicules, VP4, are proteolytically dissociated into two subunits, VP8* and VP5*, as a result of the presence of proteases. VP8* is responsible for specific binding to the target cell and VP5* is responsible for membrane penetration and entry into the cell [19]. During infection, a series of molecular transformations in the outer layer cleave this layer from the rest, so that only the transcriptionally active inner capsid, known as the DLP, enters the cytosol [20].

The conventional classification system is based on genome composition and immunological reactivity of three structural proteins: VP6, VP7 and VP4. Depending on the VP6 protein, they can be classified into nine species: groups A to I [14,21,22] with a recent proposal for a J species [23]. Groups A, B, C and H RV have been associated with AGE in humans and animals. The most common of the four, RVAs, are responsible for more than 90% of infections in humans and animals. Four RVA subgroups can be distinguished (SGI, SGII, SGI1II and SG nonI-nonII) based on reactivity patterns with two subgroup-specific monoclonal antibodies directed to VP6 [18]. Within these four subgroups, SGII is most frequently found in humans, while SGI is most common among those infecting animals [22].

The two external capsid proteins VP7 and VP4 trigger production of neutralizing antibodies, which can confer protective immunity against viral infection. Serotypes/genotypes are defined by these two outer capsid antigens: VP7 determines serotype G (a glycoprotein) and VP4 serotypes P (by sensitivity to proteases), with P[4], P[6] and P[8] being the most common. Overall, there are five prevalent genotypes: G1P[8], G2P[4], G3P[8], G4P[8] and G9P[8] [24], which represent more than 90% of strains circulating worldwide. Currently, a total of 36 G and 51 P types have been described globally [25], among which the G1P[8] genotype was the most common in the pre-vaccine era. However, after vaccine introduction the diversity of circulating genotypes has increased considerably in different countries, including South Korea, the United Kingdom and Brazil, with genotype G2P[4] reaching greater predominance [26,27,28]. In addition, several studies report G12P[8] as among the genotypes with most increased prevalence in the post-vaccine era [29]. Even so, it is estimated that the P[8] genotype is responsible for more than 80% of human infections [30]. Within this genotype, four different lineages have been described phylogenetically: P[8]-lineage I, P[8]-lineage II, P[8]-lineage III and P[8]-lineage IV [31,32]. Furthermore, recent advances in molecular characterization techniques have revealed that the P[8] genotype can be divided antigenically into different subtypes: P[8]a (composed of lineages I and III) and P[8]b (also called OP354-like, composed of lineage IV) [33]. Most strains circulating in the world belong to subtype P[8]a, while subtype P[8]b is less common. In general, use of the terms serotype or genotype depends on whether immunological detection (MAb-ELISA and cross neutralization assay) or nucleic acid-based (sequencing, RT-PCR or oligonucleotide probe hybridization) methods have been used [18]. In addition to this system, there is also another one based on the genotype of the NSP4 protein, a viral enterotoxin [34].

In April 2008 the Rotavirus Classification Working Group (RCWG) developed a complete genome classification system based on the nucleotide sequence for the RVA group. This new classification system assigns a specific genotype to each of the 11 genomic segments of a particular RV strain, according to established nucleotide percentage cut-off values. In this approach, the different genes comprising the RV strain genome (VP7-VP4-VP6-VP1-VP2-VP3-NSP1-NSP2-NSP3-NSP4-NSP5/6) are described using the following abbreviations: Gx-P[x]-Ix-Rx-Cx-Mx-Ax-Nx-Tx-Ex-Hx, where x represents an Arabic number starting from one, assigned on the basis of the aforementioned percentage [35]. In other words, this system assigns a number to a nucleotide sequence of a gene that codes for a certain protein. Subsequently, when the gene sequence of a particular strain is analyzed, if this gene is 85% similar to a previously described sequence (that has already been assigned a certain number), it is given the same value. If on the other hand, if this sequence contains more than 15% difference from the defined sequences, it is assigned a new number following the order of the list. Lastly, numbers associated with the VP7 and VP4 proteins are maintained. This new system has made it possible to classify the main RV constellations. Although many different RV types within group A with different P and G genotypes are able to infect humans, the extensive full genome data currently available suggest that RVA strains from only two different genotype constellations have been able to infect humans worldwide over long periods. These two constellations, known as Wa-like and DS-1-like, have been assigned the following classification: I1-R1-C1-M1- A1-N1-T1-E1-H1 and I2-R2-C2-M2-A2-N2-T2-E2-H2, respectively, not including the G and P genes. There is also a third genotypic constellation of RV with more limited spreadability known as AU-1, which has been assigned the following classification: I3-R3-C3-M3-A3/A12-N3-T3-E3-H3/H6 [36]. These constellations, although not defined by genotypes G and P, tend to have the following genotypes: G1P[8], G3P[8], G4P[8], G9P[8], G12P[6] and G12P[8] in the case of Wa-like and G2P[4] in the case of DS-1-like [37].

To sum up, this new classification system can be used not only to group different strains into constellations but also to easily identify regroupings between different strains or constellations.

## 2. HBGAs as Viral Receptors and Their Implications

Innate resistance to viral infections can be attributed to mutations in genes involved in immune response or receptor/ligand interaction. In RV, resistance to infection appears to be genotype-dependent, is mediated mainly by histo-blood group antigens (HBGAs) [38,39] and could be conditioned by microbiota composition [40]. For over 30 years, sialic acid was considered the glycoside receptor of animal RVs, yet human strains were considered independent from sialic acid [41]. The recently discovered role of HBGAs as potential receptors has significantly advanced understanding of RV diversity, evolution and epidemiology. HBGAs are expressed on the surface of target cells and act as viral receptors. Their synthesis is mediated by fucosyltransferases and glycosyltransferases under the control of the FUT 2 (secretory), FUT 3 (Lewis) and ABO (H) genes. Furthermore, the two main vaccines (Rotarix and RotaTeq) have been associated with similar susceptibility to natural infections, and since HBGA distribution varies between populations and ethnic groups, this is an important factor to take into account in the effectiveness and protectiveness of vaccines in different populations [42].

**Figure 1:**
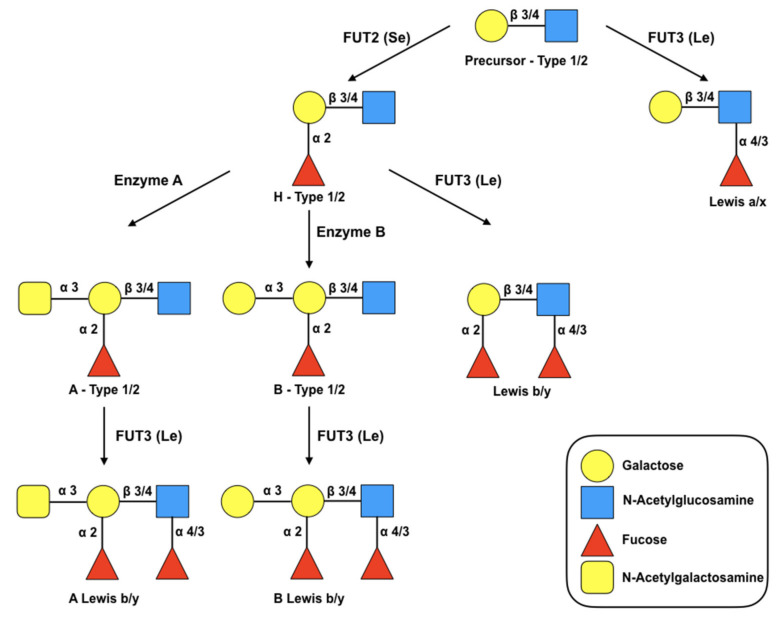
Biosynthesis route of HBGAs. These are synthesized by sequential addition of a monosaccharide to the terminal disaccharide of the precursor glycan. The figure shows the synthesis pathways starting from type 1 and 2 precursors. The glycosidic bonds are separated by a diagonal bar to specify the pathways of the different chains. The fucosyltransferases FUT2 and FUT3 drive the biosynthesis of the secretory (Se) and Lewis (Le) antigens, respectively, by catalyzing the specific addition of α-fucose residues. Enzymes A and B catalyze the specific addition of N-acetylgalactosamine and galactose respectively. Additions of glycans by enzymes A, B and FUT2 to the precursor galactose residue result in ABH HBGA. The addition of α-fucose to the N-acetylglucosamine residue by the enzyme FUT3 produces Lewis antigens (Lewis a/x, Lewis b/y, A Lewis b/y, B Lewis b/y).

HBGA synthesis occurs through sequential addition of monosaccharides to precursor molecules (disaccharides), a process shown in Figure 1. The precursor consists of a galactose molecule (Gal) bound to an N-acetylglucosamine molecule (GlcNAc) that form a disaccharide. Depending on the bond that joins both sugars, the precursors are classified into two different groups: type I (link β-1,3) or type II (link β-1,4) [41]. Secretor status is determined by the FUT 2 gene, which encodes for galactosidase 2 alpha-fucosyltransferase (adds a fucose residue in the α-1,2 position of the Gal residue), where non-secretors lack functionality in that enzyme and are consequently unable to express the H antigen [41]. The other gene that is part of this system codes for FUT3 (Lewis gene), which adds another fucose residue in position α-1,3 or α-1,4 to the GlcNAc motif of the initial precursors or H (secretory state) of type I or type II [42]. In summary, depending on the presence of recessive or dominant alleles, individuals can be secretors or non-secretors and Lewis-positive or Lewis-negative.

Multiple studies have shown interactions between HBGAs and rotavirus distal VP8* spike VP4 portion (~27 kDa, N-terminal), and it is thought that HBGAs expressed on the surface of target cells serve as viral receptors. There is evidence for P[8] and P[4] genotypes binding to the Lewis b and H type-1 (H1) antigens [43], for P[6] genotype binding to the H1 antigen [43] and for P[19] genotype binding to mucin core glycans and the type-1 HBGA precursor [44]. In addition, P[9], P[14] and P[25] genotypes bind specifically to type A antigens [45,46], whereas P[11] binds to the type-2 precursor glycan [47]. Recent studies have demonstrated by crystallography that H1 and its precursor, lacto-N-biose (LNB), bind to the VP8* P[8] protein with a two-fold increase in affinity for the H1 antigen compared to LNB, providing an explanation for lower rotavirus P[8] genotype infectivity in the non-secretor population. Moreover, LNB is a basic component of human milk, so it could be used as a possible anti-viral against RV [30].

As a consequence of the above, non-secretors are believed to be less susceptible to infection with VP8* P[6] RV. In addition, Nordgren et al. reported that VP8* P[6] preferentially infect Lewis-negative children [48], whereas Lewis-positive and secretor-positive children are more likely to be infected with VP8* P[8] [30,49]. This is particularly important considering genotype distribution across populations. For instance, in Europe and North America, it is estimated that 20% of the population are non-secretors, limiting the spread of the VP8* P[6] genotype. However, in Africa the percentage of non-secretors is much lower, hence encountering fewer impediments to expansion, which explains the abundance of the P[6] genotype on this continent (and to a lesser extent in Asia) [50]. Studies in countries such as Mali, Ghana, Kenya, Bangladesh and Vietnam have reported a high prevalence of the VP8* P[6] genotype, in some cases as high as 84.8% [51], which highlights the importance of providing protection against this genotype, particularly in LICs. The discovery of the role of these receptors, together with the genotypic distribution of different (mainly LIC) populations, could pave the way towards personalized vaccines, which can give protection against the specific genotypes that predominantly infect a certain population.

## 3. RV Vaccine Pipeline

In 1983, Bishop et al. reported that children infected with RV during the first month of life were protected against moderate-to-severe disease caused by reinfection [52]. This was the first evidence that a vaccine against RV was possible but also set certain limits for the potential vaccine, since it would not be able to prevent mild disease caused by reinfection [53]. The first vaccine to appear on the market was Rotashield (Wyeth-Lederle). However, it was withdrawn due to cases of intussusception in July 1999, only 9 months after its release into the market [54]. Intussusception is the invagination of one segment of the intestine into another, causing a medical emergency that can be deadly if left untreated. Seven years and several million deaths later, two companies (Merck and GSK) launched their vaccines on the market in 2006 (Rotarix and RotaTeq, respectively) which has led to a 90% reduction in hospitalization for diarrhea [55]. Since that date, the most widely used vaccines globally have been RotaTeq and Rotarix, although two other vaccines have more recently been developed in India: Rotavac (Bharat Biotec) and Rotasiil (Serum Institute of India) (these last two newly licensed vaccines are in use only in India (both) and Palestine (Rotavac only)). In the rest of the world, in 2018, 74 countries included Rotarix in their national immunization programs, 14 used RotaTeq and nine used both (International Vaccine Access Center (IVAC), 2020). Overall, RV vaccines have been licensed in over 130 countries and incorporated by 106 countries into their national immunization programs [56].

All these vaccines, which are pre-qualified by the WHO, are live attenuated oral vaccines given to children between 6 and 8 weeks of age. Rotarix is provided in two doses with a time lag of one month, while RotaTeq, Rotavac and Rotasiil are given in three doses.

### 3.1. Licensed Vaccines

In the following section, we will outline the main characteristics of the most widely used vaccines worldwide and those approved for use in different countries.

#### 3.1.1. Globally Licensed Vaccines

- Rotarix (RV1—GlaxoSmithKline Biologicals, Rixensart, Belgium)

Rotarix is a monovalent vaccine against the G1P[8] antigen. Studies show that in developed countries this vaccine prevents 82% of severe diarrhea cases caused by RV and accounts for about 37% of all cases of severe childhood diarrhea. However, in developing countries, it prevents only 35% of VR diarrhea cases, accounting for 17% of all cases of severe childhood diarrhea [57].

- RotaTeq (RV5—Merck and Co. Inc., Kenilworth, NJ, USA)

RotaTeq is a pentavalent vaccine containing five RV G genotypes that have been incorporated by rearrangement between human viruses and a bovine strain (bovine WC3 and human surface viral proteins of G1, G2, G3, G4 and P[8] serotypes). In countries with low mortality, it prevents 82% of VR-associated diarrhea cases. Like Rotarix, however, its effectiveness in countries with high birth rates is reduced to 41% in preventing severe VR diarrhea cases, preventing 15% of all cases of severe diarrhea [57].

- Rotavac (Bharat Biotec, India)

This monovalent vaccine against G9P [11] lacks studies demonstrating its efficacy in countries with a low infant mortality rate, and its efficacy in India (a country with a high infant mortality rate) is similar to the two previous cases. Rotavac prevents 54% of diarrhea cases in India, accounting for 16% of all cases of severe diarrhea.[57]

- Rotasiil (Serum Institute of India, India)

Rotasiil is a lyophilized pentavalent vaccine containing strains from human-bovine rearrangements with the G1, G2, G3, G4 and G9 serotypes. Its unique feature is being the first thermostable vaccine in its lyophilized format, which can maintain stability at 40° C for up to 18 months [8].

#### 3.1.2. Nationally Licensed Vaccines

These vaccines play a crucial role in facilitating affordable and available RV vaccination.

- Rotavin-M1 (POLYVAC, Hanoi, Vietnam)

This monovalent attenuated oral vaccine designed against G1P[8] is administered in two doses [58] and is licensed in Vietnam [55]. It was produced to make the country self-sufficient and is given in two doses at 2 and 4 months of age.

- Lanzhou lamb (Lanzhou Institute of Biological Products, China)

This is an oral live attenuated vaccine consisting of the G10P[15] genotype, which was first isolated in 1985 and is administered in a single dose followed by annual boosters [59]. It is approved for sale and use in China [55], with recommended annual administration to children between 2 months and 3 years of age. However, as this vaccine is not included in the Chinese immunization program its coverage is rather limited [8].

In short, two main strategies have been identified for all licensed live attenuated vaccines. RotaTeq and Rotasiil consist of a RV of animal origin attenuated for host-range restriction, generated from a set of reassortant virus strains, containing VP7 G1, G2, G3, G4 and a VP4 P[8] of human origin in the case of RotaTeq, and VP7 G1, G2, G3, G4 and G9 in the case of Rotasiil. Rotarix and Rotavac consist of cultures of attenuated human RV strains.

**Figure 2:**
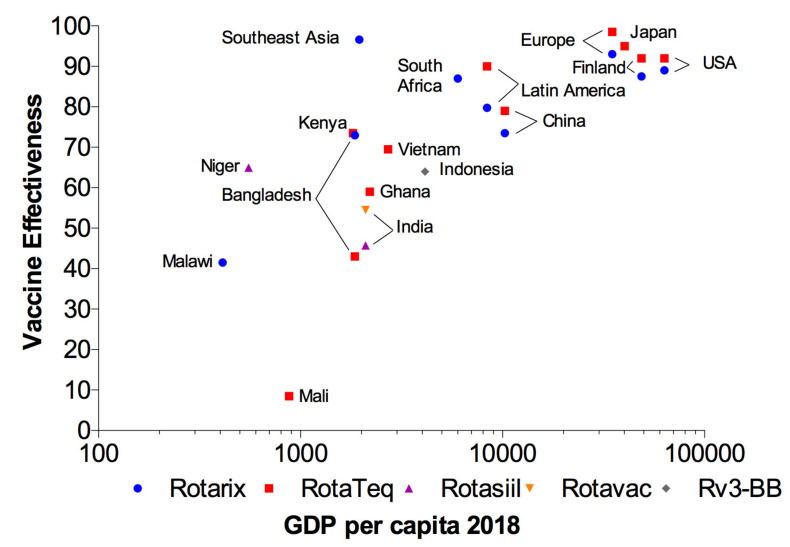
Effectiveness of oral RV vaccines in relation to a country’s per capita GDP. Effectiveness of the four main oral RV vaccines (Rotarix, RotaTeq, Rotavac and Rotasiil) is shown in countries with different gross domestic product (GDP). This Figure was generated according to world development indicators from the Worldbank and vaccine effectiveness studies [66].

These vaccines have demonstrated high protective immunity in developed countries; problematically, however, they have been shown to be less effective in LICs, where the need for them is greatest, as can be seen in Figure 2. Although the specific cause of reduced effectiveness of oral vaccines in LICs has not been demonstrated, as is the case with polio or cholera [60,61], interference caused by high titers of antibodies against RV acquired via the transplacental route or breastmilk, micronutrient deficiency, malnutrition, interfering intestinal microbiota, enteric co-infections, concomitant diseases and differences in RV epidemiology could contribute to this sub-optimal performance [62]. Another major problem with oral RV vaccines—post-marketing safety monitoring—shows these vaccines are associated with incidence of intussusception slightly increased to nearly 20 per 100,000 children, especially in the first 7 days after administration [55]. Nevertheless, other studies found no increased risk of serious adverse events, including intussusception, following vaccination [57]. It is also worth mentioning the presence of porcine circovirus (PCV) type 1 DNA in Rotarix and PCV type 1 and 2 DNA fragments in RotaTeq. To make matters worse, PCV type 1 in Rotarix was found to be infectious and capable of actively replicating in human hepatocellular carcinoma cells [63,64,65].

Rotavirus is excreted in high concentrations in the feces of symptomatic and asymptomatic individuals; indeed, it is estimated that before symptoms onset individuals are able to excrete more than 10^10^–10^11^ particles of RV per gram of feces [18], which can usually be detected in untreated sewage samples [67]. In addition, owing to inefficient sewage treatment or illegal sewage connections, RV presence is very common in different sources of environmental samples in LICs [68]. This, combined with the fact that the virus is transmitted via the fecal–oral route and that less than 100 particles are required to infect healthy individuals, opens up many sources of infection in LICs. Furthermore, some studies have shown elimination of pentavalent vaccine (Rotarix) in infant stool samples after the first or second dose, and the presence of serotypes derived from vaccine strains has been linked to cases of gastroenteritis in children. These studies indicate the possible horizontal transmission of a live attenuated vaccine and the potential environmental spread of new reassortant strains leading to development of new infections [68]. Other studies have similarly demonstrated viral elimination in stool samples at relatively short intervals after Rotarix [69] and RotaTeq [70] vaccination, which involves risk of vaccine strain transmission. In general, transmission of the attenuated vaccine strain from vaccinated to non-immunized children can lead to herd immunity, contributing greatly to the protection of a population. However, it also carries a risk of causing infection in immunosuppressed patients [71,72]. Finally, it should be noted that some live attenuated vaccines are contraindicated during pregnancy, due to risk of congenital disease in the fetus (as happened with chickenpox and rubella) [73].

Other barriers, including age restrictions on vaccine use, safety issues, the live attenuated nature and substantial costs of the vaccine, and the need for a cold chain (sometimes difficult to maintain in LICs), currently restrict the full potential for disease prevention. Research is still ongoing to overcome these limits and is now focused on implementing new oral vaccines and developing parenteral vaccines.

As a consequence of the above, it is estimated that about 83,158 of the 128,500 deaths that occurred in 2016 were preventable [74]. Other studies have determined that only 35% of children worldwide receive the vaccine, leaving more than 89 million children unprotected [75]. Put concisely, the current status of RV vaccines reflects significant progress in recent years but also reveals that much work remains to be done.

This reality is very similar to the situation with polio vaccines. Several studies in India showed that the predominant vaccine, OPV (Sabin), was less effective there than in other parts of the world, for unclear reasons. In this context, India’s national polio eradication program administered up to 10 or more doses of OPV to infants and some still developed paralysis. Another vaccine, parenteral vaccine IPV (Salk), was licensed in 1955 compared with OPV’s license in 1962 but was virtually unused as it did not produce the same herd immunity. However, subsequent studies showed that a combination of OPV and IPV could significantly increase the effectiveness of vaccination [76]. As the estimated fifth most mortal vaccine-preventable disease in children under 5 years of age [77], there is an urgent need for improvement in prevention of RV infections.

### 3.2. Vaccines under Development

**Figure 3:**
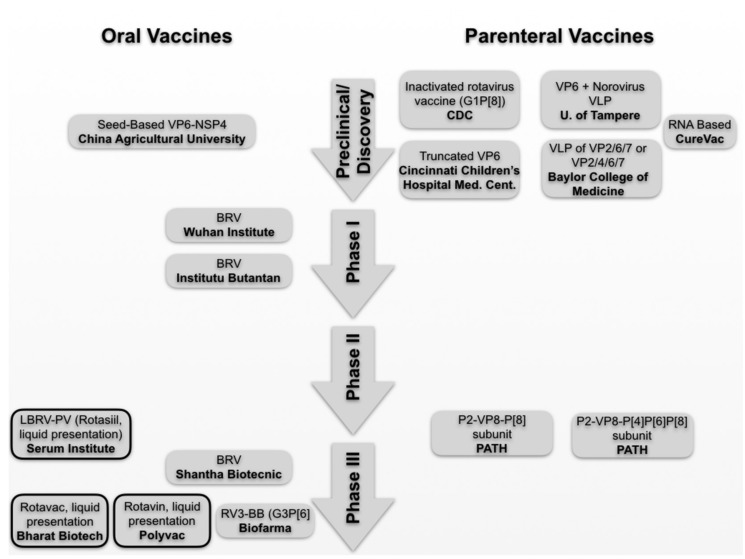
RV vaccines under development. The different vaccines are shown stratified by development phase. Oral vaccines are shown in the left column, while parenterally administered vaccines are shown on the right. The boxes with continuous lines in bold indicate licensed products pursuing new formulations.

To overcome these limitations and make the vaccine accessible to most of the child population worldwide, as well as to improve the effectiveness and duration of vaccine-induced immunity, research continues on new formulations (buffers, adjuvants, etc.); new vaccination formats (newborn doses, boosters, etc.); new oral vaccines (human neonatal vaccine RV3-BB) [78]; and, finally, new types of injectable vaccines that, since they are introduced directly into the bloodstream, avoid the intestine and thus the risk of causing intussusception. However, since the first contact with infectious RV particles occurs at the mucosal level, it is important to acquire not only systemic but also mucosal immunity. In this respect, different studies have shown that parenteral vaccines are able to induce mucosal immunity [79]. Even in RV, a vaccine administered either intramuscularly or via microneedles has been shown to induce a mucosal immunity similar to oral vaccines (and even a dose-saving effect) [80]. Furthermore, studies have been reported of parenteral vaccines formulated with components that direct trafficking to the mucosa, thus inducing expression of the reference receptor on T cells and B cells and subsequent migration to the mucosal compartments [81]. In summary, it has now been demonstrated that parenteral vaccines are capable of producing mucosal immunity. The main vaccines in development and their current phase of development are summarized in Figure 3.

#### 3.2.1. Oral Vaccines

Among new vaccines in development, the most advanced candidate is the RV3-BB vaccine (PT BioFarma, Bandung, Indonesia), an oral vaccine based on a naturally attenuated neonatal strain of the RV G3P[6]. Due to the ability of this strain to replicate in the intestine of newborns despite the presence of maternal antibodies, it is targeted for administration to infants to provide early protection against RV [8].

A noteworthy new technology that could prove particularly useful in development of new RV vaccines is the plant-based vaccine. As a first advantage, this type of vaccine does not require cold chain maintenance, as it can be transported as seeds and grown in situ, at the point of administration. Researchers from the College of Biological Sciences, University of China have developed a bivalent vaccine candidate expressing rotavirus subunits VP6 and NSP4 fused with the adjuvant B subunit of *E. coli* heat-labile enterotoxin LTB in maize seeds that successfully stimulate systemic and mucosal responses, with high titers of serum IgG and mucosal IgA antibodies [82]. Secondly, due to its minimal processing and manufacturing requirements this technology can produce a very affordable vaccine, thus favoring more globalized accessibility and therefore extending the range of protection. Although it has been known since 1990 that vaccine antigen can be expressed in plants [83], to date no plant vaccine has reached the market, and since 1990 only one product has obtained the FDA license for commercialization, as a veterinary vaccine [84]. In spite of the lack of vaccines, neutralizing antibodies have already been produced in transgenic tomatoes [85]. In short, edible plants have a promising future as important sources of human health-promoting molecules, ingestible as crude extracts or partially purified formulations.

A similar approach using modified food-related microorganisms such as yeast and *Lactococcus lactis* has been shown to confer immunity in the mouse model [86,87].

#### 3.2.2. Parenterally Administered Vaccines

Due to the previously mentioned association of oral vaccination with several risk factors such as enteropathies in the intestine, and sometimes also with reduced immunogenicity [80], most vaccines are administered parenterally in both children and adults. Intramuscular administration has proven safe and efficient in inducing an immune response. Therefore, an inactivated RV vaccine administered intramuscularly would avoid the risk factors associated with oral vaccination (including the aforementioned intussusception and diarrhea episodes associated with vaccines). In this context, several parenteral vaccines are currently in development. A possible disadvantage of parenteral vaccines may be their limited ability to induce heterologous responses in certain cases compared to live attenuated mucosal vaccines [88]. Parenterally administered vaccines avoid the need for intestinal replication of live oral vaccines. Moreover, they can be manufactured at a lower cost and are easier to transport as they are thermally stable, thus further reducing costs. Both types of vaccines have been successfully produced in other enteric infections such as polio, typhoid or cholera.

- Subunit Vaccines

Two notable examples of these vaccines are NRRV P2-VP8-P[8] subunit and P2-VP8-P[4]P[6]P[8] subunit (Figure 3), derived from fragments of the VP8* P[8] and P[4], [P6], and P[8] genotypes, respectively, from the RV spicules fused to P2 universal helper T-cell epitope derived from tetanus toxin. Studies have shown that in children who received three doses of NRRV, fecal shedding of the LORV Rotarix administered four weeks after the last dose of the trivalent P2-VP8 candidate vaccine is reduced by 57% [76]. In addition, tests conducted on South African children and infants demonstrated that those infants who had been vaccinated showed a stronger IgG response (>98% seroconversion) compared to placebo (9% seroconversion).

- Virus-Like Particles

Several vaccines are based on the use of VLP. The University of Tampere, Finland is working on a vaccine based on a VP6 subunit that incorporates norovirus-like particles (VLP) to form a combined vaccine. Preliminary results indicated that strong systemic cross-reactive NoV- and RV-antibody responses were induced in immunized mice, without mutual inhibition of the immunogenicity of either antigen in combination, indicating a viable strategy to combat two different enteric pathogens with a single vaccine shot [89]. The Cincinnati Children’s Hospital Medical Center has also incorporated the VP8* subdomain to the norovirus P (protrude) and S (shell) sub-viral particle vaccine platforms. Use of this chimera induced both rotavirus and norovirus neutralizing antibodies in mice [90]. Finally, Baylor College of Medicine has developed several VLP vaccine candidates based on the combination of VP2, VP4, VP6 and VP7 rotavirus proteins produced in a baculovirus, showing positive effect in different animals model such as mice [91], gnotobiotic piglets [92] and rabbits [93].

- Inactivated Rotavirus Vaccines

An inactivated RV vaccine (IRV) based on the CDC9 strain has also been developed for parenteral administration, which triggers a heterotypic response in the production of neutralizing antibodies. In addition, this vaccine combined with IPV is under development to achieve a co-vaccine. We found two other candidates in the early stages of development, one including an inactive G1P[8] strain, and a truncated VP4 based on the LLR strain (Lanzhou Lamb) [8]. Finally, more vaccines with little information yet available are under development, such as MT-5625, heat-activated vaccine, and viral particle vaccines [55,94].

- Nucleic Acid-Based Vaccines

Early studies from our laboratory demonstrated in mice that a DNA based vaccine was suitable and could be effective against RV infections [95]. Nucleic acid technology has been taken to the next level, and a mRNA vaccine based on the VP8* protein is now in preclinical studies (Curevac AG) [96].

## 4. RV Genotypes Included in the Vaccines

Regarding the genotypes included in vaccine formulas, as mentioned above, the VP8* P[6] genotype has taken on additional significance in developing countries due to the genotypic distribution in these populations, and moreover, because in nature almost all G genotypes have been observed in combination with P[4], P[6] and P[8] genotypes, so a combination of these three P genotypes could offer a high degree of protective immunity against all G genotypes associated with them [63]. Based on these factors, several vaccine candidates (CDC-6 vaccine) and vaccines currently in development include a P[6] genotype in their formulation (such as the trivalent parenteral non-replicating rotavirus (NRRV) vaccine group, shown in Figure 3). In addition, several studies show a substantial difference, widening over time, between current strains and the originally cultivated or sequenced strains. For instance, in the United States, the average difference between all strains with a P[8] genotype and the original Wa strain increased by 4% between 1974 and 1980, 5% 1988–1991 and 9% 2005–2013 [97]. This variation is due to point mutations and rearrangement events, and as can be observed, these events have been favored in recent years by the introduction of vaccines. Along these lines, other studies have reported significant differences between the VP8* P[8] genotype strains circulating in Belgium, where most belong to lineage III, as opposed to Rotarix (lineage I) and RotaTeq (lineage II) strains [51]. Similar results have been documented in countries such as India [98], Russia [99] and Japan [100].

## 5. Microneedle Technology

A growing tendency in recent years is skin vaccination using microneedle patches, as already reported in a study of one RV candidate [49]. These patches have a number of advantages: they do not need a cold chain, can be applied by minimally trained personnel (of special consideration in developing countries) and do not generate hazardous biological waste [101]. A variety of studies have proposed this as a viable vaccination method, given that large-scale production of these vaccines could be profitable and the thermostability of this product has been demonstrated for dry vaccines [102,103,104,105]. These vaccines have already been studied for different diseases, such as rabies, influenza, hepatitis B and polio, showing the main advantage that the vaccine content is administered directly to antigen-presenting cells in the epidermis, which may result in a reduced number of required doses, a lower dose of antigen needed and improved vaccine-induced protection [106]. Studies in murine models against other diseases show that these microneedles can induce comparable immunogenicity against influenza as intramuscular injection, using only one-thirtieth of the current dose [105]. Furthermore, this study also shows that these micro-patches could be stored at 23 °C for at least 6 months.

## 6. Conclusions

As outlined above, the number of RV deaths has reduced significantly since the introduction of vaccines. However, due to their lack of accessibility and reduced effectiveness in LICs, many potentially preventable deaths still occur. Different lines of research are therefore underway, which include new formulations, as exemplified by the multiple parenteral vaccines currently in development to avoid cases of intussusception caused by use of oral vaccines.

Finally, this article has reported two novel technologies whose introduction into RV vaccines could solve several of the principal problems. First, vaccines using micro-patches for administration do not require qualified personnel for their administration and have high thermostability; and second, the use of edible plants as vaccines would produce cheaper vaccines, with enhanced accessibility worldwide and easier administration. Of these two proposals, the latter seems a more distant reality that requires further research.
